# Association between oral health and oral health-related quality of life in patients before hip and knee endoprosthesis surgery: a cross-sectional study

**DOI:** 10.1186/s12903-022-02650-z

**Published:** 2022-12-14

**Authors:** Gerhard Schmalz, Fabian Fenske, Florentine Reuschel, Markus Bartl, Laura Schmidt, Szymon Goralski, Andreas Roth, Dirk Ziebolz

**Affiliations:** 1 Department of Cariology, Endodontology and Periodontology, University of Leipzig, Liebigstr. 12, Leipzig, 04103 Germany; 2 Department of Craniomaxillofacial Surgery, University of Leipzig, Leipzig, Germany; 3 Department of Orthopaedics, Trauma and Plastic Surgery, University Hospital Leipzig, Leipzig, 04103 Germany

**Keywords:** Dental care, Endoprosthesis, Oral health, Oral related quality of life, Response shift

## Abstract

**Objectives:**

Aim of this cross-sectional study was the assessment of oral health-related quality of life (OHRQoL) health-related quality of life (HRQoL), oral health behaviour and oral health status in patients before hip and knee endoprosthesis (EP) surgery. Moreover, associations between OHRQoL, HRQoL and oral health should be examined.

**Methods:**

Consecutive patients before hip and/or knee EP implantation were recruited and referred to the dental clinic for oral examination including: number of remaining teeth, dental findings (DMF-T-Index), periodontal condition (periodontal treatment need, Staging/Grading) and temporomandibular joint screening. OHRQoL was assessed by the German short form of oral health impact profile (OHIP G14), HRQoL by short-form 36 survey.

**Results:**

Hundred and sixty two patients with a mean age of 66.80 ± 11.10 years were included, which had on average 18.22 ± 8.57 remaining teeth and a periodontal treatment need of 84.5%. The OHIP G14 sum score revealed a median of 1 (mean: 2.7 ± 4.4, 25–75th percentile: 0–4) and its dimension oral function of 0 (mean: 0.8 ± 1.8, 25–75th percentile: 0–1), what was also found for psychosocial impact (median: 0, mean: 1.4 ± 2.6, 25–75th percentile: 0–2). The OHIP G14 sum score and both dimensions were significantly associated with mental component summary (*p* < 0.01). A higher number of remaining teeth as well as remaining molars/premolars were associated with lower OHIP G14 sum score (*p* = 0.02). This was also found for the dimension oral function (*p* < 0.01).

**Conclusion:**

Patients prior to hip and knee EP had an unaffected OHRQoL, although they had an insufficient oral health. Individuals before EP implantation need increased attention in dental care, fostering information, sensibilization and motivation of the patients.

## Introduction

As one of the most relevant elective orthopaedic procedures worldwide, surgical replacement of hip and knee with an endoprosthesis (EP) is frequently used for pain reduction and to increase functional capacity of advanced joint diseases [1–3]. This, however, brings a potential risk of complications for this elective treatment, especially infections of the EP, affecting morbidity, mortality and quality of life in affected patients [1]. For those infectious complications, the oral cavity or an oral focus can be a potential source, respectively [4–7]. Accordingly, oral health, oral health behaviour and patient´s perception on oral health issues is one potential field of interest to affect the risk of complications in patients with EP.

Generally, patients prior to orthopaedic joint replacement by an EP suffer from pain and large functional restrictions, largely affecting their health-related quality of life (HRQoL) [8–10]. Therefore, those patients have a high level of suffering, affecting their whole life and daily living. Thereby, both, physical as well as mental components of HRQoL are perceived as affected by the respective patients [8]. As a sub-aspect of the whole HRQoL, the oral health-related quality of life (OHRQoL) represents the quality of life impairment related to the oral cavity, including teeth and periodontium, mouth and dentures [11]. Thus, oral diseases like periodontitis, caries and tooth loss were reported to affect both OHRQoL and HRQoL [12–14]. However, patients with chronic general diseases were reported to show an altered perception of their OHRQoL; it has been found that patients undergoing renal replacement therapy, suffering from rheumatic diseases or after organ transplantation often have a non-affected OHRQoL, which appeared irrespective of their worse physical oral health [15–17]. Thereby, a kind of response shift has been concluded, explaining that patients have a reduced perception of oral diseases compared to healthy individuals, because of their general disease burden [17].

This aspect would be of particular interest in the group of patients prior to EP implantation; although discussed controversially, those patients could have a certain risk of developing an infectious complication of the EP because of an oral focus [4–7]. Accordingly, increased dental attention and related early rehabilitation and sufficient maintenance care were recommended by recent literature [18, 19]. In contrast, the prevalence and severity of oral diseases in patients prior to joint replacement seems high [20, 21]. A recent cohort study applied a special dental referral concept for patients before EP surgery and revealed that one third of them had at least one potential focus for EP infection [22]. In summary, the oral health situation of those patients seem inappropriate and to be an area needing improvement, especially against the background of the potential infectious risks related to the oral cavity.

Taken together, the insufficient oral situation of patients prior to EP on the one, and their enormous general disease burden on the other hand would make a response shift regarding OHRQoL probable. This would partly explain the reduced dental care situation of the patients and would identify the patient perspective, especially their sensibilisation and motivation for oral health issues as a determining approach. Until now, no studies are available, which considered the OHRQoL prior to EP implantation. Therefore, the current study had two aims. The first objective was the evaluation of OHRQoL, HRQoL and oral health status in a cohort of patients prior to hip and knee EP surgery. Second, the study aimed in the assessment of potential associations between OHRQoL and HRQoL, oral hygiene behaviour and oral health status of the patients. It was hypothesized that the patients would show an OHRQoL within the German reference range for orally healthy individuals [23]. Moreover, it was expected that OHRQoL would be associated with HRQoL, but not with oral health status.

## Methods

This current cross-sectional study was performed in full accordance with the Declaration of Helsinki and was reviewed and approved by the ethics committee of the Medical Faculty of Leipzig University (No: 116/20-ek). All participating individuals gave their written informed consent for the current study, after a verbal and written information on the study.

### Patients

A cohort of patients prior to EP surgery at the Department of Orthopaedics, Trauma and Plastic Surgery, University Hospital Leipzig, Germany was recruited for the current study between April 2020 and July 2021. Within a dental referral concept between the department of orthopaedics and the dental clinic, patients underwent a full dental examination and risk stratification prior to EP implantation, as described previously [22]. Thereby, patients were asked for their voluntary participation in the study. For this current examination, the following inclusion criteria were formulated: age between 18 and 89 years, status prior to EP surgery of the first EP (hip or knee). In addition, the exclusion criteria were as follows:

- Worse general health conditions, which would not allow an oral examination.
- Cognitive and/or psychiatric diseases (e.g., severe dementia).
- Acute indication of joint replacement, e.g., in context of traumata.
- In need of care in nursing home.
- Insufficient German language abilities, which did not allow answering the questionnaires.
- Previous EP surgery.

The sample size of the current study was estimated based on previous studies on OHRQoL of patients with different chronic general diseases [15–17]. A specific sample size calculation was not performed; to reach a reasonable patient group, especially in sub-group comparisons, a minimum number of 100 participants was targeted. Nevertheless, it was aimed to include as many patients as possible during the study period.

Based on a medical history, general information were assessed (age, gender, smoking) alongside with several information on oral hygiene behaviour. All included individuals received two different questionnaires:

### Questionnaires

#### Oral health impact profile (OHIP G14)

To evaluate the OHRQoL of the participating patients, the German short version of Oral Health Impact Profile (OHIP G14) was applied [23, 24]. This validated questionnaire consists of 14 functional and psychosocial impacts that participants have experienced in the previous month resulting from complaints with their teeth, mouth or dentures. In answering the OHIP G14 questions, five different answering possibilities were available: very often = “4”, fairly often = “3”, occasionally = “2”, hardly ever = “1”, and never = “0”. The result of the OHIP G14 is a score, which ranged between “0” (all questions answered with “never”) and “56” (all questions answered with “very often”); thus, higher values reflect a reduced/worse OHRQoL. For analysis of the OHIP G14 findings, the sum score of OHIP G14 values as well as the two dimensions “oral function” and “psychosocial impact” were analyzed [25].

#### Short form-36 health survey (SF-36)

To measure the HRQoL of included patients, the 36 items-consisting SF-36 was applied [26], which has been used in the German translated form for this current study [27]. For analysis, the scales physical functioning, role functioning/physical, general health, energy/fatigue, pain, social functioning, emotional well-being and mental well-being were calculated. Moreover, the two higher-ordered clusters physical component summary (PCS) and mental component summary (MCS) were formed for further analysis. All values are shown on a scale between 0 and 100, whereby higher values indicate better HRQoL.

### Oral examination

The full oral examination was performed once under standardized conditions in the dental clinic by two experienced and calibrated dentists as described before [22]. The calibration process included the independent examination of the same patients by the two examiners prior to the study onset. Those patients were generally healthy individuals. The results of the examinations were compared and the process was repeated with different patients until the overlap between the two examiners was higher than 80% (kappa > 0.8). Thereby, the investigation included dental, periodontal and temporomandibular examinations. Dental health was evaluated visually by mirror and probe, whereby the number of remaining teeth as well as the presence of caries with cavitation of the tooth surface (D-T) were recorded. If patients had at least one carious tooth (D-T > 0), dental treatment need was rated. The periodontal examination included a full periodontal status with a measurement of periodontal probing depth (PPD), clinical attachment loss (CAL) and bleeding on probing (BOP) at six measurement points each tooth with a respective periodontal probe (PCP 15/11.5B6, Hu-Friedy, Chicago, IL, USA). According to the available staging and grading matrix [28], the periodontitis diagnosis was formulated and periodontal treatment need was defined (PPD > 4 mm in more than two sextants and/or more than two independently teeth).

- Stage I: interdental CAL max. 1–2 mm.
- Stage II: interdental CAL max. 3–4 mm.
- Stage III: interdental CAL max. ≥ 5 mm, periodontitis-related tooth loss ≤ 4 teeth.
- Stage IV: interdental CAL max. ≥ 5 mm, periodontitis-related tooth loss ≥ 5 teeth.
- Grade A: bone loss/age < 0.25.
- Grade B: bone loss/age 0.25–1.0 and/or smoking < cigarettes/day and/or diabetes mellitus with HbA1c < 7.0%.
- Grade C: bone loss/age < 1.0 and/or smoking ≥ 10 cigarettes/day and/or diabetes mellitus with HbA1c ≥ 7%.

Furthermore, the periodontal inflamed surface area was estimated as presented in literature [29].

Additionally, patients were screened with regard to temporomandibular disorders (TMD). Thereby, the presence of any complaints and conspicuous findings of the temporomandibular joint was recorded according to Ahlers and Jakstat [30]. The clinical examination was complemented by a panoramic radiograph. Based on the whole oral health findings, the risk for an EP infection with a potential oral focus was estimated based on a risk stratification, as described previously in detail [22]. Based on the presence of treatment need and/or potential oral foci (e.g. caries involving the pulp, severe periodontal treatment need (e.g., suppuration, endo-perio-lesion), apical radiolucency (= sign for chronical infection/inflammation), (partly) retained teeth with pericoronal inflammation) patients were either categorized as low, moderate or high-risk patients, respectively.

### Statistical analysis

The analysis was performed using SPSS for Windows (version 24.0, SPSS Inc., US). The OHRQoL as well as HRQoL and oral health findings were described descriptively. To detect internal consistency of the sum score and sub-scale scores, Cronbach’s alpha was calculated. Furthermore, associations between OHIP G14 sum score and the dimensions psychosocial impact and oral function were evaluated. Therefore, the median of the sum score/dimensions was used to distribute between higher and lower OHRQoL, respectively.

Kolmogorov–Smirnov test showed that none of the metric variables was normal distributed (*p* < 0.05). Accordingly, non-parametric tests for non-normal distributed samples were applied. Comparing two independent variables, Mann–Whitney-U test was used. Categorical and nominal data were analysed by chi-square or Fisher test, respectively. The significance level was set at *p* < 0.05.

## Results

### Patients

In the current study, 162 patients with a mean age of 66.80 ± 11.10 years were included. About one quarter of the patients smoked. About half of patients stated to regularly visit the dentist for professional tooth cleaning (48.8%) and less than one third (31.7%) stated to perform interdental cleaning (Table 1).

**Table 1:** Patient characteristics and oral hygiene behaviour of included individuals

	Patients prior to EP (n = 162)
Gender (male in %)	48.8%
Age in years (mv ± sd)	66.80 ± 11.10
Smoking habits %	
Smoker	24.7%
Non-smoker	75.3%
Regular dental visits %	
Yes	74.5%
No	25.5%
Regular professional tooth cleaning %	
Yes	48.8%
No	51.2%
Tooth brush %	
Manual	64.2%
Powered	35.8%
Interdental cleaning	
Yes	31.7%
No	68.3%

*EP* Endoprosthesis; *mv* Mean value; *sd* Standard deviation

### Oral health and treatment need

On average, the patients had 18.22 ± 8.57 remaining teeth. The periodontal treatment need was 84.5%, while more than half of the patients had a stage IV periodontitis (57.4%). More than one third (36.5%) were in the high-risk group for EP infections with potential oral origin, indicating that this amount of patients had at least one dental focus (Table 2).

**Table 2:** Results of the oral examinations

Parameter	Patients prior to EP (n = 162)
D-T (mv ± sd)	0.36 ± 0.84
Number of remaining teeth (mv ± sd)	18.22 ± 8.57
Number of remaining molars/premolars (mv ± sd)	8.88 ± 5.45
Number of remaining front teeth (mv ± sd)	9.34 ± 3.72
BOP % (mv ± sd)	24.05 ± 16.67
Periodontal treatment need %	84.5%
Dental treatment need %	30.2%
Periodontitis stage %	
I	0
II	0.9%
III	41.7%
IV	57.4%
Periodontitis grade %	
A	0
B	79.6%
C	20.4%
PISA in mm² (mv ± sd)	292.93 ± 258.59
TMD screening %	
Conspicious	21%
Inconspicious	79%
Risk class for EP infection with oral origin	
Low	23.2%
Moderate	40.3%
High	36.5%

*Mv* Mean value; *sd* Standard deviation; *D-T* Number of carious teeth; *BOP* Bleeding on probing; *PISA* Periodontal inflamed surface area; *TMD* Temporomandibular disorders, Stage I: interdental CAL max. 1–2 mm, Stage II: interdental CAL max. 3–4 mm, Stage III: interdental CAL max. ≥ 5 mm, periodontitis-related tooth loss ≤ 4 teeth, Stage IV: interdental CAL max. ≥ 5 mm, periodontitis-related tooth loss ≥ 5 teeth, Grade A: bone loss/age < 0.25, Grade B: bone loss/age 0.25–1.0 and/or smoking < cigarettes/day and/or diabetes mellitus with HbA1c < 7.0%, Grade C: bone loss/age < 1.0 and/or smoking ≥ 10 cigarrettes/day and/or diabetes mellitus with HbA1c ≥ 7%, periodontal treatment need: PPD > 4 mm in more than two sextants and/or more than two independently teeth, dental treatment need: D-T > 0

### OHIP G14 values

The distribution of answers is shown in Table 3. It is conspicuous, that the vast majority answered the questions with 0 (never). The dimension oral function had a median of 0 (mean: 0.8 ± 1.8, 25–75th percentile: 0–1), what was also been found for the dimension psychosocial impact (median: 0, mean: 1.4 ± 2.6, 25–75th percentile: 0–2). The OHIP G14 sum score revealed a median of 1 (mean: 2.7 ± 4.4, 25–75th percentile: 0–4). Cronbach’s alpha values were 0.96 for OHIP G14 sum score, and for the dimensions oral function and psychosocial impact, values of 0.93 and 0.94 were determined, respectively.

**Table 3:** Results of the different questions within the German short form of the oral health impact profile (OHIP G14)

Question [n]	Point score OHIP G14				
	Never (Rating 0)	Rarely (Rating 1)	Sometimes (Rating 2)	Often (Rating 3)	Very often (Rating 4)
Trouble pronouncing	145	16	0	1	0
Taste worsened	150	9	0	1	2
Life less satisfying	139	15	3	3	2
Difficult to relax	129	20	12	1	0
Feeling of tension	137	20	4	0	1
Interrupting meals	145	11	5	1	0
Uncomfortable to eat	137	10	13	0	2
Short tempered	143	12	4	2	1
Difficulty performing jobs	139	16	5	0	2
Unable to function	146	13	3	0	0
Embarrassed	143	14	4	1	0
Diet unsatisfactory	148	12	1	0	1
Oral pain	128	24	8	2	0
Sense of uncertainty	130	20	12	0	0

*OHIP* Oral health impact profile

### SF-36 values

The findings of the SF-36, reflecting the HRQoL, are given in Table 4. The physical component summary (PCS) showed a mean of 28.0 ± 8.2 and the mental component summary (MCS) was on average 52.4 ± 10.6.

**Table 4:** SF 36 of the included patients

Parameter	Patients prior to EP (n = 162)
SF-36 physical functioning (mv ± sd)	33.4 ± 23.8
SF-36 role functioning/physical (mv ± sd)	29.2 ± 19.4
SF-36 general health (mv ± sd)	52.5 ± 18.1
SF-36 energy/fatigue (mv ± sd)	48.8 ± 18.7
SF-36 pain (mv ± sd)	30.3 ± 20.9
SF-36 social functioning (mv ± sd)	68.6 ± 25.8
SF-36 emotional well-being (mv ± sd)	68.9 ± 43.1
SF-36 mental well-being (mv ± sd)	69.4 ± 17.9
PCS (mv ± sd)	28.0 ± 8.2
MCS (mv ± sd)	52.4 ± 10.6

*mv* Mean value; *sd* Standard deviation; *SF-36* Short form 36 survey; *PCS* Physical component summary; *MCS* Mental component summary

### Associations between OHIP G14 and general parameters as well as HRQoL

The OHIP G14 sum score showed a significant association with the MCS, whereby better OHRQoL was associated with better HRQoL (MCS 56.4 ± 9.1 vs. 47.3 ± 10.4, *p* < 0.01). Further associations for the OHIP G14 sum score were not confirmed (Table 5). Similarly, the dimensions psychosocial impact (*p* < 0.01) and oral function (*p* < 0.01) were significantly associated with MCS (Table 6).

**Table 5:** Association between OHIP G14 sum score with general parameters, oral hygiene and health-related quality of life

Parameter	OHIP G14 sum score		
	≤ 1	2+	*p*-value
Smoking	29.3%	18.6%	0.14
Age	66.57 ± 11.35	67.10 ± 10.83	0.75
Gender male	52.2%	44.3%	0.34
Regular professional tooth cleaning	48.9%	48.6%	0.99
Interdental cleaning	37.4%	24.3%	0.09
PCS	28.7 ± 8.6	27.2 ± 7.7	0.37
MCS	56.4 ± 9.1	47.3 ± 10.4	**< 0.01**

*PCS* Physical component summary; *MCS* Mental component summary; *OHIP* Oral health impact profile; significant results (*p* < 0.05) are highlighted in bold

**Table 6:** Association between psychosocial impact and oral function with general parameters, oral hygiene and health-related quality of life

Parameter	Psychosocial impact			Oral function		
	0	1+	*p*-value	0	1+	*p*-value
Smoking	27.6%	19.3%	0.26	27.1%	18.2%	0.31
Age	66.50 ± 11.20	67.33 ± 10.98	0.62	66.40 ± 11.12	67.86 ± 11.09	0.36
Gender male	50.5%	45.6%	0.62	50%	45.5%	0.72
Regular professional tooth cleaning	47.6%	50.9%	0.74	47.4%	52.3%	0.60
Interdental cleaning	32.7%	29.8%	0.73	33.3%	27.3%	0.57
PSC	28.7 ± 8.5	26.8 ± 7.5	0.25	28.5 ± 8.5	26.7 ± 7.4	0.40
MCS	55.6 ± 9.3	46.5 ± 10.5	**< 0.01**	54.7 ± 9.8	46.6 ± 10.5	**< 0.01**

*PCS* Physical component summary; *MCS* Mental component summary, significant results (*p* < 0.05) are highlighted in bold

### Associations between OHIP G14 and oral health

A higher number of remaining teeth (19.33 ± 8.69 vs.16.75 ± 8.24, *p* = 0.02) as well as remaining molars/premolars (9.72 ± 5.58 vs. 7.77 ± 5.16, *p* = 0.02) were associated with lower OHIP G14 sum score (Table 7). This was also found for the dimension oral function; thereby, number of remaining teeth (19.32 ± 8.55 vs. 15.30 ± 7.99, *p* < 0.01), number of remaining molars/premolars (9.64 ± 5.58 vs. 6.86 ± 4.67, *p* < 0.01) as well as number of remaining front teeth (9.68 ± 3.60 vs. 8.41 ± 3.90, *p* = 0.01) were associated with oral function. Moreover, patients with worse OHRQoL in the dimension oral function had significantly more periodontitis stage IV (47.4% vs. 83.3%, *p* < 0.01). For the dimension psychosocial impact, no associations with oral health were confirmed (*p* > 0.05, Table 8).

**Table 7:** Association between OHIP G14 sum score with oral health parameters

Parameter	OHIP G14 sum score		
	≤ 1	2+	*p*-value
D-T	0.33 ± 0.68	0.40 ± 1.01	0.67
Number of remaining teeth	19.33 ± 8.69	16.75 ± 8.24	**0.02**
Remaining molars/premolars	9.72 ± 5.58	7.77 ± 5.16	**0.02**
Remaining front teeth	9.61 ± 3.65	8.97 ± 3.80	0.14
BOP in %	22.83 ± 13.67	25.62 ± 19.96	0.81
Periodontitis stage IV	49.2%	68.1%	0.12
Periodontitis grade C	14.8%	27.7%	0.15
PISA in mm²	289.28 ± 234.20	297.67 ± 289.76	0.69
TMD conspicuous	20.7%	21.4%	0.46
Risk class high	33%	41.2%	0.11

*D-T* Number of carious teeth; *BOP* Bleeding on probing; *PISA* Periodontal inflamed surface area; *TMD* Temporomandibular disorders; *OHIP* Oral health impact profile; significant results (*p* < 0.05) are highlighted in bold

**Table 8:** Association between psychosocial impact and oral function with oral health parameters

Parameter	Psychosocial impact			Oral function		
	0	1+	*p*-value	0	1+	*p*-value
D-T	0.26 ± 0.60	0.54 ± 1.13	0.23	0.36 ± 0.86	0.34 ± 0.78	0.96
Number of remaining teeth	18.56 ± 8.98	17.61 ± 7.79	0.24	19.32 ± 8.55	15.30 ± 7.99	**< 0.01**
Remaining molars/premolars	9.17 ± 5.78	8.35 ± 4.86	0.34	9.64 ± 5.58	6.86 ± 4.67	**< 0.01**
Remaining front teeth	9.38 ± 3.80	9.26 ± 3.58	0.39	9.68 ± 3.60	8.41 ± 3.90	**0.01**
BOP in %	22.60 ± 16.52	26.23 ± 16.85	0.22	24.75 ± 17.33	22.21 ± 14.94	0.55
Periodontitis stage IV	53.8%	62.8%	0.51	47.4%	83.3%	**< 0.01**
Periodontitis grade C	15.4%	27.9%	0.15	15.4%	33.3%	0.06
PISA in mm²	257.63 ± 223.02	346.28 ± 299.54	0.15	300.28 ± 245.58	273.81 ± 293.37	0.25
TMD conspicuous	17.1%	28.1%	0.17	20.3%	22.7%	0.67
Risk class high	35%	39.3%	0.71	33.3%	45.2%	0.19

*D-T* Number of carious teeth; *BOP* Bleeding on probing; *PISA* Periodontal inflamed surface area; *TMD* Temporomandibular disorders, significant results (*p* < 0.05) are highlighted in bold

## Discussion

### Summary of the main results

The OHIP G14 sum score showed a median of 1 point. An oral health association was predominantly found in the dimension oral function; the number of remaining teeth and periodontitis stage were the factors with an association to oral function. With regard to HRQoL, the MCS was associated with OHIP G14 sum score as well as to both investigated dimensions.

### Comparison with published data

Generally, the topic of oral health alongside with OHRQoL in patients treated with EP is of certain importance and clinical relevance. Accordingly, the current study aimed in the assessment of oral health, oral health behaviour and patient´s perception on oral health issues as one potential field of interest to affect the risk of complications in patients with EP. Thereby, this is the first study on OHRQoL of patients prior to EP implantation. Generally, it was aimed to gain a better understanding of the patient perspective, to reveal potential explanations for the insufficient oral health situation of those individuals. Against the background of recent literature, which showed a high periodontal burden in patients before EP surgery [20–22], the current study found comparably high periodontal treatment need and disease severity. This is not surprising, because the patients were part of a cohort, which has already been examined separately, before [22]. It is known that periodontal disease can affect OHRQoL [12, 31]. Moreover, tooth loss, which was also present in the current study, has been reported to be an important influential factor on OHRQoL [14, 32]. Therefore, one might have expected that the OHRQoL of the current study´s cohort would be reduced. However, confirming the previously formulated hypothesis of the current study, there was a median in OHIP G14 sum score of 1 point, which reflects an unaffected OHRQoL. A previous study by John et al. (2004) showed that fully dentate, orally healthy German individuals should have a median sum score of ≤ 2 and partly dentate patients should have a median sum score ≤ 4 points [23]. Thus, the patients in the current study showed a sum score within this range, reflecting an unimpaired OHRQoL. This is contradictory to their enormous prevalence of oral diseases.

Previous studies on several groups of patients with general diseases found quite similar results; patients undergoing chronic haemodialysis were found to present an unaffected OHRQoL, especially when the time under dialysis was long, what lead to worse physical oral health [33]. Patients with rheumatoid arthritis, which suffered from chronic pain, as this was present for the patients prior to EP in the current study, did also show a nearly unaffected OHRQoL, although their oral disease burden was high [34]. Those patients are not completely comparable to the current study´s cohort, but in the absence of comparable literature for patients before EP, those comparisons support the interpretation of the data. It seems like there is a similar phenomenon as concluded in a systematic review on OHRQoL after organ transplantation: it has been described that patients with a severe chronic disease undergo a response shift, i.e. a reducing perception of the importance of oral health issues against their general disease burden [17]. This approach goes back to the original response shift theory by Sprangers and Schwarz (1999), which described the accommodation of a patient as chronically ill, whereby a contrast or difference is perceived less intensive [35]. Similarly, the patients before EP implantation appear to perceive oral diseases as less important, what unfortunately seems to lead to an insufficient oral health situation. This is particularly troublesome, as there is a potential role of oral inflammation in the development of EP infections [4–7]. Those perioprosthetic infections are very serious complications after EP implantation; the caused morbidity, alongside with reduced quality of life and pain is enormous [36]. The consequences of EP infection are mostly comprehensive, reaching from revision surgery to amputation, but also systemic infections, sepsis and a risk of mortality is apparent [37]. Therefore, the prevention of EP infections has a very high priority in the pre- and postoperative care.

The current study found the MCS to be associated with OHRQoL. This supports the response shift theory in this context, because primarily the emotional burden resulting from the chronic pain appears to influence the OHRQoL. Generally, the HRQoL of the included patients prior to EP was reduced, what is in line with available literature [1–3]. In addition, a previous study on patients with heart failure did also show that OHIP G14 sum score was associated with HRQoL, including both PCS and MCS [38]. Therefore, an impact of general disease-related issues on oral health perception appears reasonable for patients with chronical diseases. This current study confirmed this for patients prior to EP surgery for the first time. Regarding oral health, the number of remaining teeth did affect OHRQoL, primarily in the dimension oral function. Literature showed that tooth loss is related with OHRQoL [7, 32]. Thereby, especially the number of remaining functional occlusal pairs was found as a strong predictor of OHRQoL [32]. This supports the finding of the current study that the number of remaining molars was associated with both, oral function and OHIP G14 sum score. Additionally, periodontitis stage was associated with the dimension oral function. Within the staging matrix, tooth loss is a main issue leading to the evaluation as stage IV periodontitis [25]. Therefore, the association with periodontitis stage IV might be explained by the effect of tooth loss on OHRQoL. Altogether, only end-stage oral diseases (tooth loss as final consequence of dental and/or severe periodontal diseases) appear to affect OHRQoL of patients before EP; this would also support the response shift, whereby only large oral health concerns (loss of teeth) are considered as annoying by the patients. This was similarly found in a cohort of patients with rheumatoid arthritis, in which tooth loss was associated to OHRQoL, alongside with disease specific factors [34].

Taken together, the current study showed that patients prior to EP implantation / surgery perceive their OHRQoL as not affected, although they show a high prevalence of oral diseases. Therefore, increased attention should be paid on those individuals in dental context, fostering information, sensibilization and motivation of the patients. Thereby, patient-centred approaches should be applied, e.g. in form of visual metaphors and comprehensive information, as introduced previously [39].

### Strengths and limitations

This is the first clinical study, which investigated OHRQoL alongside with oral health and HRQoL in a cohort of patients prior to EP. The cohort was large, whereby 162 patients could be included. Valid instruments and a comprehensive examination were applied to reveal various clinically relevant data. Especially the OHIP G14 is a highly valid and recommendable tool for application in research [40]. The internal consistency of the instrument within the current study was excellent, what is comparable to other patient groups, e.g. with rheumatic diseases [41]. Several limitations require consideration in the interpretation of the findings. This was a cross-sectional, monocentric study. Accordingly, the transferability and generalizability of the findings as well as any longitudinal effects, especially after EP implantation cannot been estimated, yet. No control group was recruited for comparison; however, using national reference values [23] is a common procedure and suitable for comparison of OHIP G14 data, which has already been performed previously [33, 34]. Regarding OHRQoL outcome data, the recruitment in a study on oral health issues might be an influential factor on the perception responses of patients. Although this is a quite general limitation, this needs to be recognized in the interpretation of the data. Especially, several recent global problems might also affect the results of the current study and must be kept in mind; on the one hand, the COVID-19 pandemic is hardly influencing daily life and thus might have affected the results of the current study. On the other hand, structural changes, e.g. related to global warming might affect the perception of oral health concerns. The cohort in the current study was a quite heterogeneous sample, whereby different co-morbidities and medications could have been of certain relevance, but were not considered in the current study. To better understand the changes in oral health perception related to EP surgery, a longitudinal design would be required. Thereby, patients should be examined during follow-up prior and after surgery. This would be of interest for future studies in the field.

## Conclusion

Patients prior to hip and knee EP showed an OHRQoL within the German reference range for orally healthy individuals, although they had an insufficient oral health. The OHRQoL was associated with tooth loss and MCS. Therefore, patients before EP implantation need increased attention in dental care, fostering information, sensibilization and motivation of the patients.

## Data Availability

The datasets used and/or analyzed during the current study are available from the corresponding author on reasonable request. The data are not publically available, because of the psedonymisation and data protection guidelines according to the ethics approval.
